# The Dental Aspect of Epilepsy

**Published:** 1901-04-15

**Authors:** H. C. Brown

**Affiliations:** Gallipolis, Ohio


### THE DENTAL ASPECT OF EPILEPSY.

BY H. C. BROWN, D.D.S., GALLIPOLIS, OHIO.

Read at the Ohio State Dental Society.

I am practising in Gallipolis, where the State hospital for epileptics is located. I might state incidentally that this was the first State institution founded in America for the treatment of epileptics, and that now there are something like one thousand patients under treatment. Epilepsy is a disease with which few people are acquainted, and few realize how common it is. The superintendent, Dr. H. C. Rutter, estimates that there are more than four thousand so afflicted in this State. All who come into contact with these people realize the great need of a dentist's attention, but they have no dentist. The patients pay very little attention to their teeth. If anything is done for them in the institution it is usually an extraction; they send the patients first to the asylum druggist, and when he fails, we get them. They are a few patients, of families which are well to do, that are sent to the different dentists of the city.

Although they are a class of patients whose teeth require a great deal of attention, yet they get very little. The principal treatment in the hospital is the administration of the bromides, and they are given in very large doses. The patients have fetid breath and highly inflamed gums. The mucous membrane is not very sensitive, however; if it were they would suffer a great deal more from pain than they do.

It would seem that dental irritation must play an important part in causing the epileptic attacks. I have operated upon patients and kept informed of them afterwards, where I know that the removal of some badly decayed or ulcerated teeth had brought about an improvement in the patient and the attacks have been less severe and not so frequent as before.

Such patients require a great deal of attention. The last thing they do before passing into one of their attacks is to give a sudden inward gasp of breath, and it has happened that things which were present in their mouths have been drawn down into the larynx. When this happens they may die from suffocation before the trouble is discovered. Therefore too much care in operating can not be practised. This will also indicate the necessity of their retaining the natural teeth, if possible. Partial plates and bridges which are likely to work loose are dangerous. If a patient wears a plate the first thing the attendant does at the beginning of an attack is to try and remove it.

There is no doubt they do suffer with odontalgia to a certain extent, but not so much as other people would, as their sensibility is seemingly reduced to a low point.

One peculiarity of these people is that when one goes for attention to the dentist, all who are acquainted with and know of it become immediately affected in a similar manner. When this happens they are usually sent to the druggist, who extracts their teeth. This is the druggist connected with the hospital. I do not know what effect the bromides have on the osseous system, but I know they do reduce the vitality of the patient and the teeth seem to be much softer than those not taking treatment for epilepsy.

